# Budget Impact of Rivaroxaban for the Prevention of Thromboembolic Events after Hip or Knee Replacement in Spain

**DOI:** 10.36469/9824

**Published:** 2016-01-28

**Authors:** Xavier Granero Xiberta, Antonio Murcia, José Ricardo Troncoso Durán, Claudio Gómez Zubeldia, Aleix Llorac Moix, Ruth Graefenhain De Codes, Maria Giovana Ferrario, Luis Lizán

**Affiliations:** 1 Hospital Universitari Germans Trias i Pujol, Badalona, Spain; 2 Hospital General Universitario Santa María del Rosell, Cartagena, Spain; 3 Hospital Povisa, Vigo, Spain; 4 Hospital Santa Ana de Motril, Motril, Granada; 5 Bayer HealthCare, Barcelona, Spain; 6 Outcomes ’10, Castellón Spain

**Keywords:** antithrombotic prophylaxis, total hip replacement, total knee replacement, major orthopedic surgery, rivaroxaban, enoxaparin, budget impact

## Abstract

**Background:** Anti-thrombotic prophylaxis is routinely used in patients undergoing elective total hip or knee replacement (THR or TKR) to reduce the risk of venous thromboembolism (VTE). In Spain, pharmacological prophylaxis is performed with low-molecular-weight heparin, enoxaparin being the most commonly used. Rivaroxaban is an oral antithrombotic drug that has shown superior efficacy and similar safety profile compared to enoxaparin regimens in randomized clinical trials. The aim of the study was to estimate the budget impact of increasing the use of rivaroxaban with respect to enoxaparin in the prophylaxis of VTE in patients undergoing elective THR or TKR.

**Methods:** A budget impact analysis was conducted in order to estimate the economic cost from an increase of rivaroxaban use versus enoxaparin by 10%, 20%, and 30% over the 3 years of the time horizon (2015, 2016, and 2017) for the THR and TKR populations. Data related to rate of thromboembolic events, major bleeding events and use of resources (local or general anesthesia and nurse care after surgery) were obtained from the Xarelto® for VTE Prophylaxis After Hip or Knee Arthroplasty (XAMOS) study, an international, non-interventional, observational, open-label study in unselected patients undergoing THR or TKR surgery in routine practice. The study included a total of 17 701 patients from 252 centers in 37 countries, including Spain, Italy, France and United Kingdom, among others. Two cohorts where considered (patients undergoing THR or TKR) with two arms (patients treated with rivaroxaban or enoxaparin). The Spanish patients enrolled in the XAMOS study were 262 with THR and 538 with TKR. Thromboembolic events, major bleeding rates and health care resources were considered from both the international and the Spanish population. Health care resources including pharmacologic prophylaxis, anesthesia and nurse care costs (Euros 2014) were estimated from the Spanish National Healthcare System (NHS) perspective. The annual cost associated with each cohort was estimated based on the mean cost per patient and the estimated distribution of use of rivaroxaban or enoxaparin in the base case scenario and alternative scenario (increase of rivaroxaban use) over the 3 years. A one-way sensitivity analysis was conducted to evaluate the effect that the uncertainty of the input parameters may have on the results of the impact budget.

**Results:** The difference in cost per patient undergoing THR or TKR with rivaroxaban versus enoxaparin was -€140.69 including event rates and resource use from the Spanish XAMOS population, and -€110.54 when considering event rates and resource use from the multinational XAMOS population (including but not limited to European [Spain, France, Italy, United Kingdom, Portugal, etc.], American [Canada, Mexico, Colombia, Venezuela, etc.], Asian [China, etc.] and Australian countries). In the analysis per cohort (THR or TKR), the impact of increasing the use of rivaroxaban in the THR cohort, was -€1106, -€2875, and -€5607 for 2015, 2016, and 2017, considering the data from the Spanish XAMOS population, and -€869, -€2259, and -€4405 considering the data from the multinational population. Considering the TKR cohort, the impact was -€2271, -€5904, and -€11 513, and -€1784, €4639, and -€9046, respectively.

**Conclusions:** The present analysis shows that, according to effectiveness data from the XAMOS study (Spanish and multinational cohorts), an increase in the usage of rivaroxaban in VTE prophylaxis would lead to significant direct cost reduction in elective THR and TKR patients.

## BACKGROUND

Major orthopedic surgery such as total hip replacement (THR) or total knee replacement (TKR) is associated with 1.7-2.3% of cases with symptomatic venous thromboembolism (VTE), including deep vein thrombosis (DVT) and pulmonary embolism (PE).[1] Despite the relatively low incidence of the VTE, and its largely asymptomatic episodes, it is the first preventable hospital-related cause of death.[2]

Anti-thrombotic prophylaxis is routinely used in patients undergoing THR or TKR, decreasing the risk of VTE by 50–75%.[3,4] In Spain, this is performed with low-molecular-weight heparins (LMWHs) in approximately 90% of the patients[5], enoxaparin being the most widely used LMWH.[6]

Despite being effective in VTE prophylaxis, enoxaparin has relevant drawbacks. LMWH cumulatively affect the kidneys, and are associated with the risk of heparin-induced thrombocytopenia.[7] From a practical pointof-view, enoxaparin is administered by subcutaneous injection, therefore, it requires patient training for post-discharge self-administration or home administration by a health care professional. Furthermore, the treatment with enoxaparin must begin 12 hours before surgery[6], and needs dose adjustment in case of extreme weight values.[7]

Conversely, new oral anticoagulants offer several advantages over enoxaparin, such as an easy route of administration as they are given orally, they are not administered before surgery, do not require routine monitoring (coagulation or platelet count) and do not need dose adjustments.[7]

Rivaroxaban is an oral direct factor Xa inhibitor that has shown superior efficacy and similar safety profile compared to enoxaparin regimens for the prevention of VTE in patients undergoing elective THR or TKR surgery in randomized clinical trials (RCTs; RECORD 1, 2, 3 and 4 trials).[8-11] However, RCT results are often biased due to the selection of the study population, therefore, observational phase IV studies can provide insights of the real-world effectiveness of therapies. The Xarelto® in the prophylaxis of postsurgical VTE after elective Major Orthopedic Surgery of hip or knee study (XAMOS) is a non-interventional, open-label cohort study conducted between January 2009 and June 2011 in 37 countries worldwide, including Spain, Italy, France, Germany, and United Kingdom, among others, which enrolled 17 701 patients undergoing hip or knee elective arthroplasty. This study compared rivaroxaban effectiveness and safety to the current standard of care (SoC) (including LMWH, unfractionated heparins, fondaparinux, dabigatran etexilate, acetylsalicylic acid and vitamin K antagonists [VKAs]).[12] The results from the XAMOS study[12] confirmed the data from the RECORD program in routine clinical practice. When compared to SoC, rivaroxaban resulted in: 1) a reduction of symptomatic thromboembolic events (arterial or VTE), 2) a reduction in major bleeding events (RECORD definition: Major bleeding was defined as bleeding that was fatal, occurred in a critical organ (e.g., retroperitoneal, intracranial, intraocular, and intraspinal bleeding) or required reoperation or extrasurgical-site bleeding that was clinically overt and was associated with a fall in the hemoglobin level of at least 2g/dL or that required transfusion of ≥2 units of whole blood or packed cells[8]) and 3) a similar rate of incidence of serious adverse events for both treatment arms.

The aim of the present study was to estimate the budget impact of increasing the use of rivaroxaban with respect to enoxaparin in the prophylaxis of VTE in patients undergoing elective THR or TKR in Spain, based on the efficacy data of the XAMOS study.

## METHODS

A budget impact model (BIM) was built in Excel 2007 for the Spanish setting. Two cohorts where considered (patients undergoing THR or TKR). Each cohort included two arms (patients treated with rivaroxaban and patients treated with enoxaparin). Thromboembolic events, major bleeding rates and health care resources were also derived from the XAMOS study, considering both the international and the Spanish population.

### Population

In the base-case scenario, data of the Spanish population from the XAMOS study was utilized, comprising 801 total patients. Two cohorts were defined according to the type of operation: 262 patients were included in the THR cohort and 538 in the TKR cohort (one patient was discarded as it was assigned neither to TKR nor to THR cohort). The number of patients submitted to surgery each year was considered to be constant.

### Treatment Cohorts

The rivaroxaban-treated arm was compared to the arm treated with the SoC (enoxaparin). Although the duration of the drug treatment was determined case by case by the attending physician, a mean duration of 30 days was assumed for all patients in this model, independently of the antithrombotic drug and localization of surgery.

### Event Rates and Healthcare Resource Use

In the base-case scenario, the event rates (thromboembolic events and major bleedings) and healthcare resource use from the Spanish population of the XAMOS study were used to calculate the budget impact. A second estimation was performed using data from the whole (multinational) XAMOS population.[13] Event rates and healthcare resource use associated with rivaroxaban or SoC treatment after either hip or knee arthroplasty are reported in Table 1.

**Table 1.** Rate of Events and Use of Health Care Resources associated with Rivaroxaban and Standard of Care (i.e. enoxaparin) in the Spanish and Multinational Cohort of the XAMOS Study

| Event or Healthcare Resource Type | | Spanish Population | | Multinational Population | | |
|---|---|---|---|---|---|---|
| | | Rivaroxaban | SoC | Rivaroxaban | SoC | Reference |
| Thromboembolic event | | 0.20% | 0.80% | 1.16% | 2.00% | 13 |
| Anesthesia | General | 18% | 23% | 43.20% | 41.80% | 13 |
| | Local | 82% | 77% | 56.80% | 58.20% | 13 |
| Major bleeding (EMA Guideline) | | 0.70% | 1.30% | 0.40% | 0.34% | 13 |
| Post-discharge treatment | Home | 3.70% | 8.20% | 2.60% | 6.90% | 13 |
| | Healthcare facility | 0.2% | 2.60% | 1.20% | 2.30% | 13 |

EMA: European Medicines Agency

### Costs

Direct costs were considered and expressed in Euros of the year 2014. The unit costs included in this evaluation were pharmacological costs, costs of thromboembolic events (as a weighted average (6:4) of the cost of the PE and DVT), costs of general and local anesthesia, major bleeding events and post-discharge treatment administration by a nurse (at home or health care facility) (Table 2).

**Table 2.** Unit Costs included in the Budget Impact Analysis

| | Type of Cost | Definition | Unit Cost (€) | Reference |
|---|---|---|---|---|
| Pharmacy cost | Rivaroxaban | Price to Public Daily cost of Xarelto® 10mg | 3.03 | 17 |
| | Enoxaparin | Price to Public average daily cost of Clexane® 40 mg (4000 UI) | 5.14 | 17 |
| Thromboembolic event | | Cost per event calculated as a weighted average (6:4) of the cost of the pulmonary embolism (Diagnosis-related Group; DRG78 ) and deep vein thrombosis (DRG128) | 3373.04 | 18 |
| Anesthesia | General | Unit cost of general anesthesia | 207.21 | 19 |
| | Local | Unit cost of local anesthesia | 51.80 | 19 |
| Major bleeding | Unit cost | 3718.68 | 5 | |
| Post-discharge treatment administration by a nurse | Home | Nurse for administration of post-discharge antithrombotic therapy | 16.30 | 19 |
| | Healthcare facility | | 7.12 | 19 |

### Scenarios

In the first year of the analysis (2015), the percentage of rivaroxaban and enoxaparin use in the basecase scenario was defined at 30% and 70%, respectively. This assumption was based on a previously published budget impact for the Spanish NHS of the prevention of VTE in patients undergoing total hip or knee replacement,[5] which considered all available treatments and validated by a Spanish experts panel. The rivaroxaban versus enoxaparin proportion was therefore calculated by discarding the rest of the treatments and expressing it on a 100% basis the market shares of rivaroxaban and enoxaparin. Utilization of rivaroxaban for subsequent years was assumed to increase by 30% yearly on the rate of the previous year (from 30% in 2015 to 39% in 2016, and 50.70% in 2017).

The alternative scenario was defined assuming a hypothetical increase of rivaroxaban use with respect to the base-case scenario (10% in 2015, 20% in 2016, and 30% in 2017). Table 3 shows the percentages of usage considered for THR and TKR patients over the 3 years in both scenarios.

**Table 3.** Percentage of Use of Rivaroxaban and Enoxaparin over 3 Years for both Hip and Knee Replacement Cohorts in the Base-case and Alternative Scenario

| Drug | Utilization Share (%) | | |
|---|---|---|---|
| | **2015** | **2016** | **2017** |
| **Base-case scenario** | | | |
| **Rivaroxaban** | 30.00 | 39.00 | 50.70 |
| **Enoxaparin** | 70.00 | 61.00 | 49.30 |
| **Alternative scenario** | | | |
| **Rivaroxaban** | 33.00 | 46.80 | 65.91 |
| **Enoxaparin** | 67.00 | 53.20 | 34.09 |

### Sensitivity Analysis

A one-way sensitivity analysis (OWSA) was performed in order to identify which variables induce a significant variation in the total costs when their values change. The variables included in the sensitivity analysis were the pharmacological costs of rivaroxaban and enoxaparin, the percentage of use of rivaroxaban, the rate of events and the health care resources associated with both treatments.

## RESULTS

### Mean Cost per Patient

The estimated mean cost per patient treated either with rivaroxaban or with enoxaparin for the prophylaxis of thromboembolic events after hip or knee replacement surgery in Spain is shown in Table 4. It was calculated as average cost weighted on the incidence or percentage use of the event or resource. The mean cost included pharmacological costs (rivaroxaban or enoxaparin for 30 days), the cost per thromboembolic event (pulmonary embolism and deep vein thrombosis), the cost of anesthesia (local or general), the cost per major bleeding event, and the cost of 30 days post-discharge treatment administration by a nurse (at home or health care center). Using the event rate data from the Spanish population, the total mean cost per patient was estimated at €221.97 for the rivaroxaban and €362.66 for the enoxaparin arm, both for the THR and the TKR cohorts. Using event rate data from the multinational population, including but not limited to European (Spain, France,

Italy, United Kingdom, Portugal, etc.), American (Canada, Mexico, Colombia, Venezuela, etc.), Asian (China, etc.) and Australian countries, a total mean cost per patient was estimated at €279.12 for the rivaroxaban and €389.66 for the enoxaparin arm.

**Table 4.** Estimated Mean Cost per Patient Treated with either Rivaroxaban or Enoxaparin for the Prophylaxis of Thromboembolic Events after Hip or Knee Replacement Surgery in Spain using Event Rate Data from either the Spanish or the Multinational XAMOS Cohort

| Cost | Pharmacy (€) | Thromboembolic event (€) | Anesthesia (€) | Major bleeding (€) | Post-discharge treatment administration by a nurse (€) | Total (€) |
|---|---|---|---|---|---|---|
| **Spanish Population** | | | | | | |
| Rivaroxaban | 90.90 | 6.75 | 79.77 | 26.03 | 18.52 | 221.97 |
| Enoxaparin | 154.14 | 26.98 | 87.54 | 48.34 | 45.65 | 362.66 |
| Difference | -63.24 | -20.23 | -7.77 | -22.31 | -27.13 | -140.69 |
| **Multinational Population** | | | | | | |
| Rivaroxaban | 90.90 | 39.13 | 118.94 | 14.87 | 15.28 | 279.12 |
| Enoxaparin | 154.14 | 67.46 | 116.76 | 12.64 | 38.65 | 389.66 |
| Difference | -63.24 | -28.33 | 2.18 | 2.23 | -23.38 | -110.54 |

Based on the data of the Spanish population of the XAMOS observational study, and according to the base-case scenario, rivaroxaban was associated with a reduced risk of VTE and major bleeding risk.[13] This resulted in a reduced cost of -€20.23 and -€22.31 per patient treated with rivaroxaban with respect to enoxaparin for VTE and major bleeding, respectively. On the other side and according to the same study, in the rivaroxaban arm, fewer patients needed general anesthesia compared to the enoxaparin arm, and fewer patients needed post-discharge nurse visits for treatment administration, resulting in a difference of -€7.77 and -€27.13 per patient, respectively. Finally, the lower price-to-public acquisition cost of rivaroxaban determined a treatment cost difference of -€63.24, while the total cost per patient difference was -€140.

On the other hand, when considering the event rates from the multinational population of the XAMOS study to calculate the cost estimation, patients treated with rivaroxaban were still associated to a smaller cost compared to patients treated with enoxaparin. However, the cost difference was smaller (-€110/patient approximately) compared to the cost estimated from the Spanish population event rates. This was mainly due to a lower rate of VTE in the Spanish population treated with rivaroxaban compared to that of the multinational.

### Total Costs of the THR and TKR Cohorts

According to the THR cohort which included 262 patients, the total cost was calculated considering the mean cost per patient described above, which was estimated from the Spanish and the multinational XAMOS study population data. The total cost of increasing the use of rivaroxaban versus enoxaparin by 10%, 20%, and 30% with respect to the base-case scenario (see Table 3) in years 2015, 2016, and 2017 in VTE prophylaxis regarding the THR Spanish and multinational population is presented in Figure 1 and Figure 2, respectively.

### Total Costs of the THR and TKR Cohorts

According to the THR cohort which included 262 patients, the total cost was calculated considering the mean cost per patient described above, which was estimated from the Spanish and the multinational XAMOS study population data. The total cost of increasing the use of rivaroxaban versus enoxaparin by 10%, 20%, and 30% with respect to the base-case scenario (see Table 3) in years 2015, 2016, and 2017 in VTE prophylaxis regarding the THR Spanish and multinational population is presented in Figure 1 and Figure 2, respectively.

**Figure 1.** THR Cohort Costs 2015-2017, Spanish Population; THR Cohort Total Costs 2015-2017 in the Basecase and alternative Scenarios in Spain Estimated on the Basis of Event Rates from the Spanish Population of the XAMOS Study

| | 2015 | 2016 | 2017 |
|---|---|---|---|
| Reference scenario (increase in 30% yearly on the rate of previous year) | 83,959 € | 80,642 € | 76,329 € |
| Alternative scenario (increase in rivaroxaban use: 10%, 20%, 30%, with respect to the reference scenario) | 82,853 € | 77,767 € | 70,722 € |

THR: total hip replacement

**Figure 2.** THR Cohort Costs 2015-2017, Multinational Population; THR Cohort Total Costs 2015-2017 in the Base-case and alternative Scenarios in Spain estimated on the Basis of Event Rates from the Multinational Population of the XAMOS Study

| | 2015 | 2016 | 2017 |
|---|---|---|---|
| Reference scenario (increase in 30% yearly on the rate of previous year) | 93,402 € | 90,795 € | 87,407 € |
| Alternative scenario (increase in rivaroxaban use: 10%, 20%, 30%, with respect to the reference scenario) | 92,533 € | 88,536 € | 83,002 € |

THR: total hip replacement

Regarding the TKR cohort, 538 patients were included. Considering the mean cost per patient described above and estimated from the Spanish and multinational XAMOS population data applied to this cohort, the total cost of increasing the use of rivaroxaban versus enoxaparin by 10%, 20%, and 30% with respect to the base-case scenario (Table 3) in years 2015, 2016, and 2017 in VTE prophylaxis in the Spanish and multinational population regarding TKR is presented in Figure 3 and Figure 4, respectively.

**Figure 3.** TKR Cohort Costs 2015-2017, Spanish population; TKR Cohort Total Costs 2015-2017 in the Basecase and alternative Scenarios in Spain Estimated on the Basis of Event Rates from the Spanish Population of the XAMOS Study

| | 2015 | 2016 | 2017 |
|---|---|---|---|
| Reference scenario (increase in 30% yearly on the rate of previous year) | 172,405 € | 165,593 € | 156,737 € |
| Alternative scenario (increase in rivaroxaban use: 10%, 20%, 30%, with respect to the reference scenario) | 170,134 € | 159,689 € | 145,224 € |

TKR: total knee replacement

**Figure 4.** TKR Cohort Costs 2015-2017, Multinational Population; TKR Cohort Total Costs 2015-2017 in the Base-case and alternative Scenarios in Spain estimated on the Basis of Event Rates from the Multinational Population of the XAMOS Study

| | 2015 | 2016 | 2017 |
|---|---|---|---|
| Reference scenario (increase in 30% yearly on the rate of previous year) | 191,795 € | 186,443 € | 179,484 € |
| Alternative scenario (increase in rivaroxaban use: 10%, 20%, 30%, with respect to the reference scenario) | 190,011 € | 181,804 € | 170,439 € |

TKR: total knee replacement

## Budget Impact

The impact of increasing the use of rivaroxaban versus enoxaparin in VTE prophylaxis in patients with THR or TKR was calculated considering the event rates and resource use from the Spanish population of the XAMOS study. The impact of increasing the use of rivaroxaban versus enoxaparin in VTE prophylaxis in the THR cohort was -€1106, -€2875, and -€5607 in the years 2015, 2016, and 2017, respectively. According to the TKR cohort, the impact of increasing the use rivaroxaban versus enoxaparin in VTE prophylaxis was -€2271, -€5904, and -€11 513 in the years 2015, 2016, and 2017, respectively.

When considering the event rates from the multinational population, the impact of increasing the use rivaroxaban versus enoxaparin in VTE prophylaxis of the THR cohort was -€869, -€2259, and -€4405 in the years 2015, 2016, and 2017, respectively. According to the TKR cohort, the impact was -€1784, -€4639, and -€9046 in the years 2015, 2016, and 2017, respectively.

Table 5 summarizes the budget impact of yearly increasing the utilization of rivaroxaban by 10%, 20% and 30% (alternative scenario) in patients with THR in Spain, considering the event rates from the Spanish and the multinational cohort.

**Table 5.** Increasing Budget Impact when increasing Rivaroxaban Utilization by Year in THR Patients in Spain

| | | **Budget Impact in THR per Year** | | |
|---|---|---|---|---|
| | | **2015** | **2016** | **2017** |
| Alternative scenario | Increase in Xarelto® utilization with respect to reference scenario | 10.00% | 20.00% | 30.00% |
| Budget impact | Spanish cohort | -€1106 | -€2875 | -€5607 |
| | Multinational cohort | -€869 | -€2259 | -€4405 |

THR: total hip replacement

Table 6 summarizes the budget impact regarding the utilization of rivaroxaban yearly increasing by 10%, 20% and 30% (alternative scenario) in patients with TKR in Spain, considering the event rates from the Spanish and the multinational population.

These results pointed out that the higher use or rivaroxaban in VTE prophylaxis in Spain, is associated to a cost reduction compared to the current SoC.

**Table 6.** Budget Impact when increasing Rivaroxaban Utilization by Year in TKR Patients in Spain

| | | **Budget Impact in TKR per year** | | |
|---|---|---|---|---|
| | | 2015 | 2016 | 2017 |
| Alternative Scenario | Increase in Xarelto® utilization with respect to reference scenario | 10.00% | 20.00% | 30.00% |
| Budget Impact | Spanish population | -€2271 | -€5904 | -€11 513 |
| | Multinational population | -€1784 | -€4639 | -€9046 |

TKR: total knee replacement

### Sensitivity Analysis

Based on the data corresponding to the reference scenario and the first year (2015), adequate percentage variations (±10%, ±5%, and ±1%, according to the type of variable) were considered in the costs of treatments, utilization rate of treatments and in event rates, respectively, in order to run the sensitivity analysis. A one-way sensitivity analysis (Figure 5 for THR and Figure 6 for TKR) revealed that the two variables with the greatest impact on the model results were the cost of enoxaparin, followed by the cost of rivaroxaban, for both THR and TKR. This was consistent with the fact that pharmacological costs represented nearly 50% of the total costs. An increase (decrease) of 10% of the cost of enoxaparin led to an increase (decrease) in total costs of 3.02% (±€7281.36 for THR and ±€14 951.80 for TKR), whilst an increase (decrease) of 10% in the cost of rivaroxaban led to a decrease (increase) of 1.18% in total costs (±€2850.75 for THR and ±€5853.83 for TKR).

The next most influencing variable appeared to be the use of rivaroxaban, as an increase (decrease) of 5% in use, leads to a decrease (increase) in total costs of ±0.30% and ±0.62% (±€2206.15 and ±€4530.19) when applied to THR or TKR, respectively.

The variation of all variables related to event rates and resource use by ±1% had only a small influence on the budget impact results.

## DISCUSSION

Only a few economic evaluations were available in Spain comparing different antithrombotic drugs for the prevention of VTE in patients undergoing elective THR or TKR, when the analysis was performed.[14,15] Rivaroxaban has been compared to enoxaparin, resulting in a cost per patient of €257.90 versus €435.20 in THR, and €251.73 versus €336.55 in TKR (year 2013).[6] In the present evaluation over a 90-day time horizon, rivaroxaban resulted dominant over enoxaparin in terms of cost per avoided VTE, due to its higher efficacy in preventing VTE. Another evaluation compared cost and effectiveness of rivaroxaban versus enoxaparin in Spain, resulting in a difference of a mean cost per patient of -€30 and -€151 (year 2008) in THR and TKR, respectively, as well as a halved risk of symptomatic VTE and double qualityadjusted life-years gained.[16] Overall, the difference in cost per treated patient estimated in the study fell in between the estimations included in previous published studies, though considering that they were assessed with different pharmacoeconomic approaches.

When the study was conducted, only one budget impact model was already published in Spain, and compared the available therapeutic options for the prophylaxis of VTE in THR and TKR. It evaluated the introduction of an oral antithrombotic agent in the Spanish market from the Spanish NHS perspective considering a target population with all THR and TKR surgery indication (not only elective operations).[5] The mentioned evaluation considered the costs of antithrombotic drugs and the complications (VTE episodes, bleedings and the post-thrombotic syndrome) during a short term and during a 5-year period. The effectiveness of the prophylaxis was estimated using a meta-analysis. Despite the differences in the model design, Gómez-Arrayás and co-workers also concluded that the use of the new oral antithrombotic drugs would be likely to reduce the rate of VTE and bleeding compared with enoxaparin, decreasing the expenditure of Spanish NHS in VTE prophylaxis.

### Limitations

One limitation of the present study was that due to the lack of data distribution between participants with hip or knee arthroplasty in the Spanish cohort of the XAMOS study, the same rate of events and resource in both cohorts had to be assumed, therefore, the cost per patient was independent of the surgery location. Another limitation of the present analysis may be found in that effectiveness data refered to a relatively small population. Due to the number of patients in the geographical subgroup, the subanalysis of outcomes only had a descriptive nature. The sample size calculation for the overall study estimated that a sample of more than 15 000 patients was needed for appropriate statistical comparisons. The small number of patients and the expected low event rate in the geographical subgroups precluded formal statistical comparisons. However, these data may be considered a representative of the clinical practice in Spain, in elective THR and TKR, as they come from the Spanish cohort of the XAMOS 2013 observational study.

## CONCLUSIONS

The present study analyzed the economic outcomes of increasing the use of rivaroxaban for the prophylaxis of VTE in patients undergoing elective total hip or knee replacement in Spain. The analysis showed that, according to effectiveness data from the XAMOS study, an international, non-interventional, observational, open-label study in unselected patients undergoing hip or knee surgery in routine practice, rivaroxaban was more effective and less costly than the current SoC, enoxaparin, in the prevention of VTE in Spain. Therefore, an increase in the usage of rivaroxaban would lead to significant direct cost reduction in THR and TKR patients.

## CONFLICT OF INTEREST DECLARATIONS

M.G. Ferrario and L. Lizán work for an independent research entity and have received remuneration for their contribution to the project as well as for the writing of this manuscript. The sponsor of the study, Bayer Healthcare, has assumed all remuneration. A. Llorac Moix and R. Graefenhain De Codes are employees of Bayer Healthcare.

However, the authors state that the results described in this manuscript, as well as their interpretation, are a consequence of the free expression of opinion and from the agreement of the publication co-authors, and that no conflicts either for obtaining or for disclosure of such results existed.
